# Treatment of Asthma

**Published:** 1890-05

**Authors:** T. Frederick Pearse


TREATMENT OF ASTHMA.—Of the thousand and one things which have been used for this disease, nothing in my experience is equal to the nitrate of sodium. I am not fond of mixing drugs, and I therefore generally give it alone. In some cases, however, with the object of promoting sleep, I combine it with hyoscymus, and in others, again, I have found the tincture of lobelia of some additional benefit. When the nitrate of sodium first came in use I gave some large doses (ten to fifteen grains) in a case of uncomplicated asthma, which had occurred in repeated attacks for some years. The first dose made the patient so sick and faint that I could hardly induce her to repeat it; but although the second dose had a similar effect, the patient was freed from her asthmatic attacks completely, and had not had a recurrence when I last saw her, two or three years afterwards. Since then I have given it in from three to five grain doses, frequently repeated, and always with the greatest benefit. With regard to hyoscymus in this affection, as well as in other diseases, I find that the ordinary doses are of little benefit. Two drachms of the tincture or of the succus for a single dose should be prescribed, and not less than one drachm when frequently repeated. Besides having an influence over many spasmodic affections, it has a most tranquilizing influence on the mind. Given alone in asthma, it will not relieve the spasm, but in combination with the nitrate of sodium, the improved condition of the patient is sometimes simply marvelous.—T. Frederick Pearse, M. D., F. R. C. S., in the *Lancet*.

